# Supplementary material for: Heart failure medication after a first hospital admission and risk of heart failure readmission, focus on beta-blockers and renin-angiotensin-aldosterone system medication: A retrospective cohort study in linked databases
Source: PLoS One. 2020 Dec 22;15(12):e0244231. doi: 10.1371/journal.pone.0244231 (PMC7755181; doi:10.1371/journal.pone.0244231)
Supplement: S1 File — (PDF) [file pone.0244231.s001.pdf]

S1 File. Propensity scores within main groups of heart failure medication

Introduction

In the Forest-plot in Figure 2 the following hazard ratios with 95% confidence intervals are shown:

ACEI and/or ARB*	Beta-blocker	Mineralocorticoid-receptor antagonist	Diuretic
1.01 (0.96-1.06)	1.00 (0.95-1.05)	1.11 (1.05-1.16)	1.17 (1.09-1.25)

*ACEI: angiotensin-converting enzyme inhibitor; ARB: angiotensin receptor blocker

This Cox model includes the baseline characteristics age, gender, number of medications (excl. particular drug) and year of admission. The year of admission corrects for changes in prescription of heart failure medication as well as a general trend in hospital care demonstrated for example by declining length of stay.

The hazard ratios with 95% confidence intervals without application of this correction with baseline characteristics are:

ACEI and/or ARB	Beta-blocker	Mineralocorticoid-receptor antagonist	Diuretic
1.03 (0.98-1.08)	1.00 (0.95-1.05)	1.14 (1.09-1.20)	1.26 (1.18-1.34)

Calculation of the propensity score

Confounding by indication could have emerged, and we adjusted for this using propensity scores in a Cox model. Propensity scores were calculated using logistic regression analyses that included the co-medication, as a proxy for comorbidities, and baseline characteristics, i.e. age, gender, total number of unique medications and year of hospital admission. The propensity score included the medication on identifying level, in general on second level ATC group with at least 10 patients, i.e. the therapeutic subgroup. The contribution of ATC groups with so few patients resulted in a disproportionate high standard error in the calculation of the propensity score. These variables were therefore excluded from the calculation. Based on mode of action and therapeutic use, on the third level ATC group, i.e. pharmacological subgroup, were included medication used in diabetes A10, non-steroid anti-inflammatory and antirheumatic products M01, psycholeptics N05 and psychoanaleptics N06.

Comedication (ATC group)	Number of patients	Note
Stomatological preparations (A01)	31	
Drugs for acid related disorders (A02)	8785	
Drugs for functional gastrointestinal disorders (A03)	621	
Antiemetics and antinauseants (A04)	17	
Bile and liver therapy (A05)	30	
Drugs for constipation (A06)	3328	
Antidiarrheals, intestinal antiinflammatory/antiinfective agents (A07)	438	
Digestives, incl. enzymes (A09)	30	
Insulins and analogues (A10A)	2248	
Blood glucose lowering drugs, excl. Insulins (A10B)	4302	
Vitamins (A11)	1322	

Mineral supplements (A12)	1214	
Antithrombotic agents (B01)	14898	
Antihemorrhagics (B02)	176	
Antianemic preparations (B03)	2240	
Blood substitutes and perfusion solutions (B05)	96	
Cardiac therapy (C01)	10515	
Antihypertensives (C02)	522	
Diuretics (C03)	18447	a
Peripheral vasodilators (C04)	42	
Vasoprotectives (C05)	111	
Beta blocking agents (C07)	13406	b
Calcium channel blockers (C08)	4183	
Agents acting on the renin-angiotensin system (C09)	14119	c
Lipid modifying agents (C10)	8750	
Antifungals for dermatological use (D01)	360	
Emollients and protectives (D02)	99	
Preparations for treatment of wounds and ulcers (D03)	13	
Antipruritics, incl. antihistamines, anesthetics, etc. (D04)	35	
Antipsoriatics (D05)	42	
Antibiotics and chemotherapeutics for dermatological use (D06)	176	
Corticosteroids, dermatological preparations (D07)	656	
Antiseptics and disinfectants (D08)	84	
Medicated dressings (D09)	11	d
Anti-acne preparations (D10)	19	
Gynecological antiinfectives and antiseptics (G01)	23	
Sex hormones and modulators of the genital system (G03)	259	
Urologicals (G04)	1771	
Pituitary and hypothalamic hormones and analogues (H01)	15	
Corticosteroids for systemic use (H02)	1867	
Thyroid therapy (H03)	1523	
Pancreatic hormones (H04)	13	
Calcium homeostasis (H05)	12	
Antibacterials for systemic use (J01)	2696	
Antimycotics for systemic use (J02)	51	
Antimycobacterials (J04)	16	
Antivirals for systemic use (J05)	31	
Vaccines (J07)	12	
Antineoplastic agents (L01)	76	
Endocrine therapy (L02)	304	
Immunosuppressants (L04)	211	
Antiinflammatory and antirheumatic products, non-steroids (M01 excl M01AH)	689	
Coxibs (M01AH)	138	
Topical products for joint and muscular pain (M02)	11	d
Muscle relaxants (M03)	35	

Antigout preparations (M04)	1176	
Drugs for treatment of bone diseases (M05)	1115	
Other drugs for disorders of the musculo-skeletal system (M09)	410	
Anesthetics (N01)	53	
Analgesics (N02)	3160	
Antiepileptics (N03)	812	
Anti-parkinson drugs (N04)	326	
Antipsychotics (N05A)	769	
Anxiolytics (N05B)	2555	
Hypnotics and sedatives (N05C)	3784	
Antidepressants (N06A)	1851	
Psychostimulants, agents used for ADHD and nootropics (N06B)	31	
Anti-dementia drugs (N06D)	146	
Other nervous system drugs (N07)	528	
Antiprotozoals (P01)	115	
Nasal preparations (R01)	288	
Drugs for obstructive airway diseases (R03)	4511	
Cough and cold preparations (R05)	1052	
Antihistamines for systemic use (R06)	650	
Ophthalmologicals (S01)	1374	
Otologicals (S02)	49	
All other therapeutic products (V03)	176	

Notes

a. The variable diuretics (C03) was excluded from the calculation of the propensity score for diuretics. The mineralocorticoid-receptor antagonist (MRA, C03DA, 8317 patients) were included.
b. The variable beta blocking agents (C07) was excluded from the calculation of the propensity score for beta-blockers.
c. The variable agents acting on the renin-angiotensin system (C09) was excluded from the calculation of the propensity score for angiotensin-converting enzyme inhibitor (ACEI) and/or angiotensin receptor blocker (ARB). Aliskiren (C09XA02) alone or in combination with other medications (49 patients), was included.
d. For the calculation of the propensity score for diuretics, the distribution between users and non-users was unequal for the variables
    - medicated dressings (D09)
    - topical products for joint and muscular pain (M02)

    The contribution to the propensity score resulted in a disproportionate high standard error. These variables were therefore excluded from the calculation of the propensity score.

Introducing the propensity score in the Cox model

The propensity score was then included in the Cox model. The baseline characteristics age, gender, number of medications (excl. particular drug) and year of admission were excluded, as they were already in the calculation for the propensity score. The hazard ratios with 95% confidence intervals are:

ACEI and/or ARB	Beta-blocker	Mineralocorticoid-receptor antagonist	Diuretic

0.98 (0.93-1.03)	0.97 (0.92-1.02)	1.09 (1.03-1.15)	1.14 (1.06-1.22)

Trimming
Excluding patients with more extreme propensity score values will result in a higher probability of similarity between both groups, i.e. treated versus non-treated patients. We excluded patients with the highest 20% propensity scores, as well as patients with the lowest 20% propensity scores. The hazard ratios with 95% confidence intervals are:

ACEI and/or ARB	Beta-blocker	Mineralocorticoid-receptor antagonist	Diuretic
1.00 (0.94-1.07)	0.97 (0.91-1.03)	1.07 (1.01-1.14)	1.14 (1.04-1.25)

Conclusion
The statistical methods described above do not result in a notable modification of the hazard ratios.
