# Supplementary material for: Heart failure medication after a first hospital admission and risk of heart failure readmission, focus on beta-blockers and renin-angiotensin-aldosterone system medication: A retrospective cohort study in linked databases
Source: PLoS One. 2020 Dec 22;15(12):e0244231. doi: 10.1371/journal.pone.0244231 (PMC7755181; doi:10.1371/journal.pone.0244231)
Supplement: S2 File — (PDF) [file pone.0244231.s002.pdf]

S2 File. Propensity scores for ACEI versus ARB and between β-blockers

Introduction
In the Forest-plot in Figure 2 the following hazard ratios with 95% confidence intervals are shown:

ARB* relative to ACEI*	Carvedilol relative to sBBHF*
1.04 (0.97-1.12)	1.33 (1.20-1.46)

*ACEI: angiotensin-converting enzyme inhibitor; ARB: angiotensin receptor blocker; sBBHF: Selective β1-blocker with heart failure registration (metoprolol, bisoprolol and nebivolol)

This Cox model includes the baseline characteristics age, gender, number of medications (excl. particular drug) and year of admission. The year of admission corrects for changes in prescription of heart failure medication as well as a general trend in hospital care demonstrated for example by declining length of stay.
The hazard ratios with 95% confidence intervals without application of this correction with baseline characteristics are:

ARB* relative to ACEI*	Carvedilol relative to sBBHF*
1.02 (0.96-1.10)	1.30 (1.18-1.44)

Calculation of the propensity score
Confounding by indication could have emerged, and we adjusted for this using propensity scores in a Cox model. Propensity scores were calculated using logistic regression analyses that included the co-medication, as a proxy for comorbidities, and baseline characteristics, i.e. age, gender, total number of unique medications and year of hospital admission. The propensity score included the medication on identifying level, in general on second level ATC group with at least 10 patients, i.e. the therapeutic subgroup. The contribution of ATC groups with so few patients resulted in a disproportionate high standard error in the calculation of the propensity score. These variables were therefore excluded from the calculation. Based on mode of action and therapeutic use, however, on the third level ATC group, i.e. pharmacological subgroup, were included medication used in diabetes A10, non-steroid anti-inflammatory and antirheumatic products M01, psycholeptics N05 and psychoanaleptics N06.

Medication (ATC group)	Number of patients		Note
	ACEI and/or ARB*	Beta-blocker	
Stomatological preparations (A01)	13	14	
Drugs for acid related disorders (A02)	5370	4957	
Drugs for functional gastrointestinal disorders (A03)	361	298	
Antiemetics and antinauseants (A04)	9	5	a
Bile and liver therapy (A05)	18	17	
Drugs for constipation (A06)	1910	1606	
Antidiarrheals, intestinal antiinflammatory/antiinfective agents (A07)	257	224	
Digestives, incl. enzymes (A09)	19	14	
Insulins and analogues (A10A)	1499	1238	
Blood glucose lowering drugs, excl. Insulins (A10B)	2897	2354	

Vitamins (A11)	744	759	
Mineral supplements (A12)	711	590	
Antithrombotic agents (B01)	9681	8508	
Antihemorrhagics (B02)	105	107	
Antianemic preparations (B03)	1331	1157	
Blood substitutes and perfusion solutions (B05)	47	47	
Cardiac therapy (C01)	6535	5850	
Antihypertensives (C02)	315	298	
Diuretics (C03)	11648	9765	
Peripheral vasodilators (C04)	25	19	
Vasoprotectives (C05)	60	50	
Beta blocking agents (C07)	8809		b
Calcium channel blockers (C08)	2508	2001	
Agents acting on the renin-angiotensin system (C09)		7988	c
Lipid modifying agents (C10)	5791	5231	
Antifungals for dermatological use (D01)	218	179	
Emollients and protectives (D02)	54	45	
Preparations for treatment of wounds and ulcers (D03)	6	5	a
Antipruritics, incl. antihistamines, anesthetics, etc. (D04)	22	17	
Antipsoriatics (D05)	27	20	
Antibiotics and chemotherapeutics for dermatological use (D06)	98	84	
Corticosteroids, dermatological preparations (D07)	397	327	
Antiseptics and disinfectants (D08)	37	42	
Medicated dressings (D09)	8	9	d
Anti-acne preparations (D10)	14	9	
Gynecological antiinfectives and antiseptics (G01)	8	12	
Sex hormones and modulators of the genital system (G03)	165	135	
Urologicals (G04)	1024	935	
Pituitary and hypothalamic hormones and analogues (H01)	10	10	
Corticosteroids for systemic use (H02)	1052	818	
Thyroid therapy (H03)	933	740	
Pancreatic hormones (H04)	7	6	
Calcium homeostasis (H05)	4	9	
Antibacterials for systemic use (J01)	1574	1360	
Antimycotics for systemic use (J02)	25	23	
Antimycobacterials (J04)	9	10	a
Antivirals for systemic use (J05)	18	17	
Vaccines (J07)	2	5	d
Antineoplastic agents (L01)	33	31	
Endocrine therapy (L02)	174	156	
Immunosuppressants (L04)	107	106	
Antiinflammatory and antirheumatic products, non-steroids (M01 excl M01AH)	380	208	
Coxibs (M01AH)	82	56	

Topical products for joint and muscular pain (M02)	8	7	a
Muscle relaxants (M03)	19	14	a
Antigout preparations (M04)	738	698	
Drugs for treatment of bone diseases (M05)	657	560	
Other drugs for disorders of the musculo-skeletal system (M09)	264	299	
Anesthetics (N01)	26	28	
Analgesics (N02)	1837	1587	
Antiepileptics (N03)	493	454	
Anti-parkinson drugs (N04)	164	145	
Antipsychotics (N05A)	434	402	
Anxiolytics (N05B)	1536	1364	
Hypnotics and sedatives (N05C)	2251	1868	
Antidepressants (N06A)	1198	976	
Psychostimulants, agents used for ADHD and nootropics (N06B)	19	14	
Anti-dementia drugs (N06D)	84	71	
Other nervous system drugs (N07)	290	272	
Antiprotozoals (P01)	67	63	
Nasal preparations (R01)	169	147	
Drugs for obstructive airway diseases (R03)	2739	2120	
Cough and cold preparations (R05)	597	477	
Antihistamines for systemic use (R06)	383	339	
Ophthalmologicals (S01)	833	693	
Otologicals (S02)	34	21	
All other therapeutic products (V03)	80	106	

Notes

a. For the comparison sBBHF versus carvedilol the distribution between the two groups was unequal for the variables
   - antiemetics and antinauseants (A04)
   - preparations for treatment of wounds and ulcers (D03)
   - antimycobacterials (J04)
   - topical products for joint and muscular pain (M02)
   - muscle relaxants (M03)

   The contribution to the propensity score resulted in a disproportionate high standard error. Theses variables were therefore excluded from the calculation of the propensity score.
b. The variable beta blocking agents (C07) was excluded from the calculation of the propensity score for the comparison sBBHF versus carvedilol.
c. The variable agents acting on the renin-angiotensin system (C09) was excluded from the calculation of the propensity score for the comparison for ACEI versus ARB. Aliskiren (C09XA02) alone or in combination with other medications (22 patients), were included.
d. For the comparison ACEI versus ARB the distribution between the two groups was unequal for the variables
   - medicated dressings (D09)
   - vaccines (J07)

   The contribution to the propensity score resulted in a disproportionate high standard error. This variable was therefore excluded from the calculation of the propensity score.

Introducing the propensity score in the Cox model
The propensity score was then included in the Cox model. The baseline characteristics age, gender, number of medications (excl. particular drug) and year of admission were excluded, as they were already in the calculation for the propensity score. The hazard ratios with 95% confidence intervals are:

ARB* relative to ACEI*	Carvedilol relative to sBBHF*
1.05 (0.98-1.12)	1.31 (1.18-1.45)

Trimming
Excluding patients with more extreme propensity score values will result in a higher probability of similarity between both groups, i.e. treated versus non-treated patients. We excluded patients with the highest 20% propensity scores, as well as patients with the lowest 20% propensity scores. The hazard ratios with 95% confidence intervals are:

ARB* relative to ACEI*	Carvedilol relative to sBBHF*
1.04 (0.95-1.14)	1.33 (1.16-1.52)

Balance
Quintiles of propensity scores were made to test for balance between the groups.
Differences in continuous variables between patients on the one medication or the other were compared using the t-test for independent samples or the Mann-Whitney U test, as appropriate. Levene's test for equality of variances was used. Between-group differences in categorical variables were compared using the Chi-square test.
For the comparison ACEI versus ARB in the first, second and fourth quintiles, year of admission was not equal. Gender was equally distributed between both treatment groups.
For the comparison sBBHF versus carvedilol in the fourth and fifth quintiles, year of admission was not equal. In the fifth quintile age was not equal. Gender was equally distributed between both treatment groups.

Inverse probability weighting
To achieve more comparability in both treatment groups inverse probablity weighting with stabilized weights was performed. The inverse probability weighting was than included in the Cox model. The baseline characteristics age, gender, number of medications (excl. particular drug) and year of admission were excluded, as they were already in the calculation for the propensity score. The hazard ratios with 95% confidence intervals are:

ARB* relative to ACEI*	Carvedilol relative to sBBHF*
1.02 (0.96-1.10)	1.30 (1.18-1.44)

Conclusion
The statistical methods described above do not result in a notable modification of the hazard ratios.
