# Supplementary material for: Heart failure medication after a first hospital admission and risk of heart failure readmission, focus on beta-blockers and renin-angiotensin-aldosterone system medication: A retrospective cohort study in linked databases
Source: PLoS One. 2020 Dec 22;15(12):e0244231. doi: 10.1371/journal.pone.0244231 (PMC7755181; doi:10.1371/journal.pone.0244231)
Supplement: Attachment — Submitted filename: Response to reviewers 200822.docx [file pone.0244231.s003.docx]

**Response to comments**

Dear Sir,

We submitted our manuscript May 14. The valuable comments/suggestions of the editors and reviewers on our manuscript (received June 20) and our response are written down in the document “Response to Reviewers July” and “Marked Up Manuscript July”. In this document you will find a response to the reviews we received August 11.

**Reviewer Notes:**

**Reviewer #1**: The authors have addressed most comments and expanded the study limitations accordingly whenever changes were not possible to be made.

**Reviewer #3**: The authors have addressed my points.

There remains one small issue. The figures are now clearer but the details of numbers at risk and events are presented in a caption and it is not clear what time point they represent. The curves look almost hand-drawn and could perhaps also be made neater.

Response:

1. We added the time points to the captions of Figures 1-6. See lines 208, 210, 212, 214, 217 and 219.
2. To optimize clarity of the Figures, we removed the censored patients from Figures 1-6.
3. We added the following text at lines 164-166:

*To optimize clarity of the Figures, censored patients (i.e. those who reached end of follow-up without readmission) are not shown.*

4. An example of how a complete adjusted Figure looks like:
