# Supplementary material for: Heart failure medication after a first hospital admission and risk of heart failure readmission, focus on beta-blockers and renin-angiotensin-aldosterone system medication: A retrospective cohort study in linked databases
Source: PLoS One. 2020 Dec 22;15(12):e0244231. doi: 10.1371/journal.pone.0244231 (PMC7755181; doi:10.1371/journal.pone.0244231)
Supplement: Attachment — Submitted filename: Response to reviewers 201126.docx [file pone.0244231.s004.docx]

## **Response to comments**

Dear Dr. Cannatà,

Thank you for the valuable comments and suggestions of the editors and reviewers on our manuscript. In this document you will find a response to each of the comments and suggestions.

We have added an example of a Figure to show the overall lay-out.

**Requests from the editors**

1. Comments: Please ensure that your manuscript meets PLOS ONE's style requirements, including those for file naming.

Reply: We checked the PLOS ONE's style requirements and adapted our manuscript.

2. Comments: We note that you have indicated that data from this study are available upon request. PLOS only allows data to be available upon request if there are legal or ethical restrictions on sharing data publicly.

Reply: Original de-identified patient data used in this analysis were obtained from the PHARMO Database Network. The PHARMO Institute provided data to Medisch Spectrum Twente/Ziekenhuisgroep Twente, which were used to create the analytical files for the study. Medisch Spectrum Twente/Ziekenhuisgroep Twente does not have the right to provide these files to a third party. Data from this study are therefore only available upon reasonable request, for researchers who meet the criteria for access to confidential data on a patient level. The corresponding author should be informed and a formal request should be addressed to the PHARMO Institute (pharmo@pharmo.nl). Requests will be submitted to the STIZON Compliance Committee for approval, which will have to judge if the person that requests the data will be eligible to access the data.

**Reviewer #1**

Comments: In the manuscript written by Kruik-Kollöffel W. et al. the authors analyzed the relationship between HF medications prescription at discharge and the risk of heart hospitalization during follow up in a large unselected HF population. The manuscript gives us some interesting insights: beta blockers and ACE-inhibitors seem to confer a protective effect in the first months after hospitalization, but lately this favourable effect might disappear. Carvedilol compared to other selective beta-blockers seems associated to a higher risk profile. Diuretics and mineralocorticoid-receptor antagonists seem associated with a higher risk of hospitalizations.

These results are interesting, showing us that the effect of HF medications might be less convincent in unselected populations from observational registries than in randomized clinical trials.

Reply: we thank the reviewer for the interest in our paper and the useful comments

However some important limitations should be mentioned:

3. Comments: As the authors stated in the limitations section, lack of characterization of the presented population is a strong limitation. Heart failure is an extreme heterogeneous disease, and the efficacy drug therapy is strongly affected by the type of heart failure and etiology.

Reply: We agree on this limitation inherent to the database: lack of more detailed information on the patient or the type of HF. The databases from the PHARMO Database Network do not contain this specific information. Due to privacy legislation in the Netherlands, there was no option to retrieve this information. This information would have made our conclusions more robust. However, according to the 2016 ESC guidelines, only slightly less patients with HFpEF and HFmrEF appear to receive ACEI/ARB, BB, MRA and diuretics compared to patients with HFrEF. The within-class comparison and post-hoc analyses on time since discharge will in all likelihood not be influenced by type of HF.

We discuss this point in the Strength and limitations paragraph: “Although we did try to adjust for confounding by indication by using propensity scores as a variable in the Cox model and with IPW [17], residual, unmeasured confounding might still be present, especially as we calculated propensity scores using logistic regression analyses based on limited information of the patient and no information on HF aetiology, comorbidities, left ventricular ejection fraction or functional class (NYHA)….”. We added the following text:

*Therefore, it was not possible to investigate for example the association between the ejection fraction and readmission.*

4. Comments: The relatively low percentage of ACE-inhibitors, beta-blockers and mineralcorticoid-receptor antagonists prescription might reflect the fact that a substantial number of patients without these drugs might had HFpEF, and consequently they could have a lower risk of HF hospitalizations. This might surely be a confounding factor that attenuated the expected benefit of HF drugs and it should be better discussed by the authors.

Reply: This is an interesting point, which we investigated in our previous publication (Kruik-Kollöffel WJ et al. Effects of European Society of Cardiology guidelines on medication profiles after hospitalization for heart failure in 22,476 Dutch patients: from 2001 till 2015. Heart Fail Rev. 2019;24: 499-510, https://link.springer.com/article/10.1007/s10741-019-09777-2). Our database covered a 15-year period in which guidelines were developed and prescribing patterns followed. The percentages of patients to whom certain medications were prescribed, did not differ much from other studies, especially those in observational research.

We added the following text to the Discussion:

*The database covered a 15-year period in which guidelines developed and prescribing patterns followed. The overall relatively low percentage of prescription of disease- modifying medications might reflect the fact that a substantial number of patients without these drugs might have HF with preserved ejection fraction, and consequently they could have a lower risk of HF hospitalizations. However, the percentages of patients to whom certain medications were prescribed, did not differ much from other studies, especially those in observational research as discussed in the earlier publication [13].*

5. Comments: About the comorbidities: do the authors have data about the renal function? I might expect that the possible harmful effect of MRA and diuretics is more evident in patients with chronic kidney disease where a strict follow up of these drugs should be done.

Reply: Unfortunately, we do not have these data.

6. Comments: I suggest to add the percentage of the events in the figure 1 to 6 in addition to the number of events.

Reply: Thank you for your advice. We have added the percentages.

**Reviewer #2**

Comments: Thank you for reviewing your paper and addressing the comments of the reviewers and of the editor. In my opinion, the quality of the paper has improved and it is more informative than the previous version.

Reply: thank you for your positive comments and appreciation.
